# Reduced *C9orf72* gene expression in c9FTD/ALS is caused by histone trimethylation, an epigenetic event detectable in blood

**DOI:** 10.1007/s00401-013-1199-1

**Published:** 2013-10-29

**Authors:** Veronique V. Belzil, Peter O. Bauer, Mercedes Prudencio, Tania F. Gendron, Caroline T. Stetler, Irene K. Yan, Luc Pregent, Lillian Daughrity, Matthew C. Baker, Rosa Rademakers, Kevin Boylan, Tushar C. Patel, Dennis W. Dickson, Leonard Petrucelli

**Affiliations:** 1 Department of Research, Neuroscience, Mayo Clinic College of Medicine, 4500 San Pablo Road, Jacksonville, FL 32224 USA; 2 Department of Neurology, Mayo Clinic, 4500 San Pablo Road, Jacksonville, FL 32224 USA; 3 Department of Transplantation, Mayo Clinic, 4500 San Pablo Road, Jacksonville, FL 32224 USA; 4 Department of Cancer Biology, Mayo Clinic College of Medicine, 4500 San Pablo Road, Jacksonville, FL 32224 USA

**Keywords:** Amyotrophic lateral sclerosis, Frontotemporal dementia, *C9orf72*, Epigenetic modification, Repeat expansion, Histone methylation

## Abstract

Individuals carrying (GGGGCC) expanded repeats in the *C9orf72* gene represent a significant portion of patients suffering from amyotrophic lateral sclerosis (ALS) and frontotemporal dementia (FTD). Elucidating how these expanded repeats cause “c9FTD/ALS” has since become an important goal of the field. Toward this end, we sought to investigate whether epigenetic changes are responsible for the decrease in *C9orf72* expression levels observed in c9FTD/ALS patients. We obtained brain tissue from ten c9FTD/ALS individuals, nine FTD/ALS cases without a *C9orf72* repeat expansion, and nine disease control participants, and generated fibroblastoid cell lines from seven *C9orf72* expanded repeat carriers and seven participants carrying normal alleles. Chromatin immunoprecipitation using antibodies for histone H3 and H4 trimethylated at lysines 9 (H3K9), 27 (H3K27), 79 (H3K79), and 20 (H4K20) revealed that these trimethylated residues bind strongly to *C9orf72* expanded repeats in brain tissue, but not to non-pathogenic repeats. Our finding that *C9orf72* mRNA levels are reduced in the frontal cortices and cerebella of c9FTD/ALS patients is consistent with trimethylation of these histone residues, an event known to repress gene expression. Moreover, treating repeat carrier-derived fibroblasts with 5-aza-2-deoxycytidine, a DNA and histone demethylating agent, not only decreased *C9orf72* binding to trimethylated histone residues, but also increased *C9orf72* mRNA expression. Our results provide compelling evidence that trimethylation of lysine residues within histones H3 and H4 is a novel mechanism involved in reducing *C9orf72* mRNA expression in expanded repeat carriers. Of importance, we show that mutant *C9orf72* binding to trimethylated H3K9 and H3K27 is detectable in blood of c9FTD/ALS patients. Confirming these exciting results using blood from a larger cohort of patients may establish this novel epigenetic event as a biomarker for c9FTD/ALS.

**Electronic supplementary material:**

The online version of this article (doi:10.1007/s00401-013-1199-1) contains supplementary material, which is available to authorized users.

## Introduction

Amyotrophic lateral sclerosis (ALS) and frontotemporal dementia (FTD) are two devastating neurodegenerative diseases seen in comorbidity in up to 50 % of patients affected with either disease [23, 28]. ALS, the most frequently occurring motor neuron disease [26], is characterized by degeneration of upper and lower motor neurons leading to muscle weakness, spasticity and atrophy during mid-adulthood, with patients having a mean life expectancy of 3–5 years after disease onset [7]. Frontotemporal dementia is one of the most frequent presenile onset dementias [19], with neuronal degeneration in the frontal and temporal lobes causing a progressive deterioration of behavior, personality and/or language [24]. Recent findings demonstrate that an expanded non-coding hexanucleotide (GGGGCC) repeat in the *chromosome 9 open reading frame 72* gene (*C9orf72*, OMIM *614260) is the most common known cause of FTD and ALS, collectively referred to as c9FTD/ALS [16, 36]. Mutation carriers have several hundred to several thousand copies of the (GGGGCC) hexanucleotide repeat, as compared to 30 or less copies in non-pathogenic carriers [16]. This discovery provided further evidence that the two diseases result from defects in the same or overlapping biological pathways.

Recent studies have suggested that *C9orf72* repeat expansions may lead to RNA-mediated toxicity through the formation of RNA foci that possibly sequester RNA-binding proteins, as well as repeat associated non-ATG (RAN) translation leading to the synthesis of aggregation-prone c9RAN proteins [2, 16, 30]. Decreases in *C9orf72* mRNA levels in brain and lymphoblasts of repeat expansion carriers have also been reported [16, 22, 36], indicating that loss of C9orf72 function may also play a role in disease pathogenesis. Moreover, the finding that downregulation of the zebrafish orthologue of *C9orf72* leads to both altered morphology of motor neuron axons and locomotor deficits, and that these features are reversed upon overexpression of wild-type human *C9orf72*, further support the notion that C9orf72 loss of function is detrimental [14]. Dual involvement of RNA toxicity and loss of function have been implicated in a variety of neurodegenerative diseases caused by repeat expansions, including myotonic dystrophy, spinocerebellar ataxia (SCA) types 8 and 31, and Fragile X-associated tremor/ataxia syndromes [35].

Increasing evidence suggests that repeat expansions can impair mRNA expression through epigenetic changes resulting from variation in DNA and histone methylation, which lead to aberrant binding of the transcriptional machinery as a consequence of heterochromatin formation [1, 4, 15, 27, 32, 41]. Epigenetic changes and altered mRNA expression have previously been linked to FTD/ALS pathogenesis. For instance, aberrant epigenetic regulation of *granulin* (*GRN)*, the gene that encodes progranulin (PGRN) and is mutated in about 5–10 % of the total FTD population, causes a decrease in *GRN* mRNA expression [18]. Of interest, protein levels of DNA methyltransferases (DNMTs), which induce DNA methylation by catalyzing methyl group transfer to cytosine residues in regulatory regions, increase pre-apoptotically in mouse motor neurons [13], and DNMT1, DNMT3a, and 5-methylcytosine are all upregulated in motor neurons of ALS patients [13]. DNMT1 has been reported to function as a maintenance methyltransferase both in repeat regions and repetitive genomic sequences characterized by a higher frequency of cytosine–guanine sequences, known as cytosine–phosphate–guanine (CpG) islands, whereas DNMT3 mainly functions in CpG islands. These islands, which are normally not methylated in active genes, are usually methylated to suppress gene expression of imprinted genes [9]. An observed-to-expected CpG ratio greater than 60 % predicts CpG islands in a genomic DNA region larger than 200 bp [20]. Based on this criterion, the observed-to-expected CpG ratio is 75 % in patients carrying 700–1,600 (GGGGCC) copies. This suggests the presence of a newly formed CpG island spanning the repeat region that is potentially at risk of abnormal methylation in expansion carriers. Given that methylation of repeat sequences, as well as hypermethylation of adjacent CpG islands within promoter regions, has been shown to aberrantly influence histone methylation processes leading to gene silencing and consequently loss of protein function [1, 37], we sought to determine whether a similar disease cascade occurs in c9FTD/ALS.

To elucidate the mechanisms causing decreased expression of *C9orf72* mRNA in mutation carriers [16, 22, 36], which could lead to loss of C9orf72 function, we investigated whether perturbed epigenetic processes are involved. Our results clearly demonstrate that trimethylation of histones H3 and H4 at several lysine residues is a novel mechanism involved in decreasing the expression of *C9orf72* mRNA in expanded repeat carriers. In addition, using peripheral blood of two expanded repeat carriers, we confirmed that these epigenetic changes are easily detectable. Future studies will need to confirm blood detection of epigenetic changes in a larger cohort of c9FTD/ALS patients to validate the biomarker capacity of this novel epigenetic event.

## Materials and methods

### Standard protocol approvals, registrations, and patient consents

Protocols were approved by the Mayo Clinic Institutional Review Board and Ethics Committee on human experimentation. All participants or authorized family members gave written informed consent after which participant and family information was gathered, skin biopsies were collected, or post-mortem analyses were performed.

### Subjects

All participants in this study were recruited at Mayo Clinic Florida and were independently ascertained as having ALS and/or FTD by trained neurologists. Frontal cortices and cerebella tissues were obtained after post-mortem analyses from four ALS and six FTD patients carrying the *C9orf72* expansion, four ALS and five FTD cases without *C9orf72* expansion, and nine disease control participants (clinical information in online resource, Table 2). Skin biopsies were collected from a total of seven *C9orf72* expansion carriers, including two healthy carriers of 28 and 30 years of age, and seven participants carrying normal alleles (clinical information in online resource, Table 3). Blood samples were obtained from two ALS patients carrying the repeat expansion, and two ALS cases carrying normal alleles (clinical information in online resource, Table 4).

### Maintenance and treatment of fibroblasts

Fibroblasts were derived from skin sampled by punch biopsy on the anterior aspect of the forearm. Fibroblasts were maintained in Dulbecco’s modified Eagle’s medium (Lonza, Basel, Switzerland) supplemented with 10 % heat-inactivated fetal bovine serum (Sigma-Aldrich, St-Louis, MO, USA), 100 units/ml penicillin, and 100 μg/ml streptomycin (Gibco, Carlsbad, CA, USA) at 37 °C, in an atmosphere containing 5 % CO_2_ and 95 % air. Cells were treated with 2 μM 5-aza-2-deoxycytidine (5-AZA) or dimethyl sulfoxide (DMSO) for 6 days before they were harvested for further experiments.

### Quantitative real-time polymerase chain reaction (qRT-PCR)

Total RNA was extracted from fibroblasts using Trizol (Invitrogen, Carlsbad, CA, USA) and from brain tissue using the RNAeasy Plus Micro Kit (QIAGEN, Venlo, Limberg, Netherlands) as per manufacturer’s instructions. RNA integrity was verified on an Agilent 2100 bioanalyzer (Agilent Technologies, Santa Clara, CA, USA). cDNA was obtained after reverse transcription polymerase chain reactions (RT-PCR) using approximately 1 μg of RNA with random primers and the High Capacity cDNA Transcription Kit (Applied Biosystems, Foster City, CA, USA) as per manufacturer’s instructions. Following standard protocols, qRT-PCR was conducted in triplicates for all samples using inventoried TaqMan gene expression assays for *C9orf72* transcript variants 1 (NM_145005.5), 2 (NM_018325.3) and 3 (NM_001256054.1) (Hs00376619), *C9orf72* transcript variants 2 and 3 (Hs00945132), *C9orf72* transcript variant 1 (Hs00331877), *GAPDH* (Hs00266705), and *H19* (Hs 00262142) (Applied Biosystems) on an ABI Prism 7900HT Fast Real-Time PCR System (Applied Biosystems). Relative quantification was determined using the ∆∆Ct method and normalized to *GAPDH*.

### Droplet digital PCR (ddPCR)

RNA was isolated from brain tissue using the RNAeasy Plus Micro Kit (QIAGEN) and cDNA was obtained by reverse transcription using random primers and the High Capacity cDNA Transcription Kit (Applied Biosystems) as per manufacturer’s instructions. Reaction mixtures were prepared for droplet digital PCR using 4 μl of reverse transcribed product, 10 μl of ddPCR 2x Master Mix (Bio-Rad, Hercules, CA, USA), 1 μl of 20x Primer and TaqMan Probe assays for *C9orf72* transcript variant 1, 2 and 3 (Hs00376619), *C9orf72* transcript variant 2 and 3 (Hs00945132), *C9orf72* transcript variant 1 (Hs00331877) (Applied Biosystems), and 5 μl of nuclease-free water, per reaction mixture. The negative control contained water only. Emulsified 1 nL reaction droplets were generated using a QX100 Droplet generator (Bio-Rad) and a droplet generator DG8 cartridge (Bio-Rad) containing 20 μl of reaction mixture and 70 μl of ddPCR droplet generation oil (Bio-Rad) per well. Thirty-five microliters of the generated droplet emulsions were transferred to 96-well PCR plates which were heat-sealed using foil sheets. Target DNA amplification was performed by thermal cycling the droplet emulsions as follows: initial denaturation at 95 °C for 10 min, followed by 40 cycles of 94 °C for 30 s and 60 °C for 1 min, then 98 °C for 10 min. The fluorescence of each thermal cycled droplet was measured using the QX100 droplet reader (Bio-Rad). Data was analyzed using the QuantaSoft software (Bio-Rad) after threshold setting on fluorescence of negative controls.

### Chromatin immunoprecipitation (ChIP)

Fibroblasts grown in 10 cm culture plates at 90–95 % confluency were treated with DMSO or 5-AZA followed by incubation with 1 % formaldehyde at 37 °C for 10 min for crosslinking. Glycine (125 mM) was then added and cells were incubated at room temperature for 5 min to quench the crosslinking. Fibroblasts were washed with ice cold PBS, scraped and pelleted by brief centrifugation at 13,000×*g*. Cell pellets were resuspended in 500 μl SDS lysis buffer (EMD Millipore, Billerica, MA, USA) containing protease inhibitors and incubated on ice for 10 min. DNA was sheared by four 10 s-on/30 s-off cycles of sonication at 35 % output. 100 μl of each sample was then diluted with 900 μl ChIP dilution buffer (EMD Millipore). For pre-cleaning, the samples were incubated with 40 μl of Protein A Agarose slurry (EMD Millipore) with rotation for 1 h at 4 °C. Samples were then incubated overnight at 4 °C with 2 μg of anti-(pan)H3 (EMD Millipore) or anti-H3K9me3 (Diagenode, Denville, NJ, USA). ChIP from brain samples was performed using 50–70 mg of tissue homogenized using a tissue grinder after crosslinking and quenching, and following the same procedure used for fibroblasts with the exception that samples were incubated overnight at 4 °C with 2 μg of anti-(pan)H3 and H4 (EMD Millipore), as well as anti-H3K9me3, H3K27me3, H3K79me3, H4K20me3 (Diagenode). Overnight incubation with antibodies was followed by a 3 h incubation with 50 μl agarose slurry at 4 °C. ChIP from blood was performed using mononuclear cells isolated using standard conditions, and washed twice using HANK’S solution (Life Technologies, Carlsbad, CA, USA). DNA was sheared by six 10 s-on/30 s-off cycles of sonication at 30 % output, then subjected to the same ChIP procedure as used for fibroblast studies. However, overnight incubation at 4 °C was performed using 1 μg of anti-(pan)H3 (EMD Millipore), anti-H3K9me3, and anti-H3K27me3 (Diagenode) antibodies followed by a 3 h incubation with 25 μl agarose slurry at 4 °C. The bound complexes for the three experiments were washed for 10 min at room temperature with rotation, first with low salt immune complex wash buffer, followed by high salt and LiCl immune complex wash buffers (all from EMD Millipore), and two final washes with TE buffer. After the final wash with TE buffer, the agarose was incubated with freshly prepared ChIP elution buffer (0.1 M NaHCO_3_, 1 % SDS) for 20 min at room temperature with rotation, after which the agarose beads were discarded. After reverse crosslinking with 200 mM NaCl at 65 °C either overnight for the fibroblast and brain studies, or 6 h for the blood study, samples were treated with 0.5 μg/μl Proteinase K for 90 min at 45 °C. The eluted DNA was purified using a DNA Clean-up kit (Zymo Research, Irvine, CA, USA) for the analysis. For semi-quantitative (semi-q) PCR, forward primer 5′AGAGGGTGGGAAAAACAAAAACAC3′ and reverse primer 5′AAAACCACGAAATCGTCTTCACTT3′ were designed using PrimerSelect (DNASTAR, Madison, WI, USA). DNA amplification was conducted by PCR using 1 μl of ChIP-obtained DNA, 0.75 μl dNTP, 1.5 μl 10x standard buffer (including 1.5 mM final MgCl_2_ concentration), 0.6 μl of each primer, 10.25 μl of nuclease-free water, and 0.3 μl of Apex Taq Polymerase (Genesee Scientific) per reaction mixture, and was run on a Mastercycler proS (Eppendorf). For each fragment, a denaturation step of 5 min was first performed at 95 °C. After that, a touchdown protocol was used which consisted of an initial cycle of 20 s denaturation at 94 °C, 20 s annealing at 65 °C and 30 s elongation at 72 °C. This was followed by ten cycles in which the annealing temperature was decreased each time by 1 °C. Then 40 cycles of 20 s denaturation at 94 °C, 20 s annealing at 58 °C and 30 s elongation at 72 °C were run. A final extension at 72 °C was performed for 5 min. DNA products were electrophoresed in a 2 % agarose gel.

### Statistical analysis

We used unpaired Student’s *t* test for comparison between two sample groups with a 95 % confidence level. One-way ANOVA followed by Tukey post hoc test was used for multiple comparisons with a 95 % confidence level. We considered the difference between comparisons to be significant when *p* < 0.05 (*p < 0.05; **p < 0.01; ***p < 0.005).

## Results

**Fig. 1:**
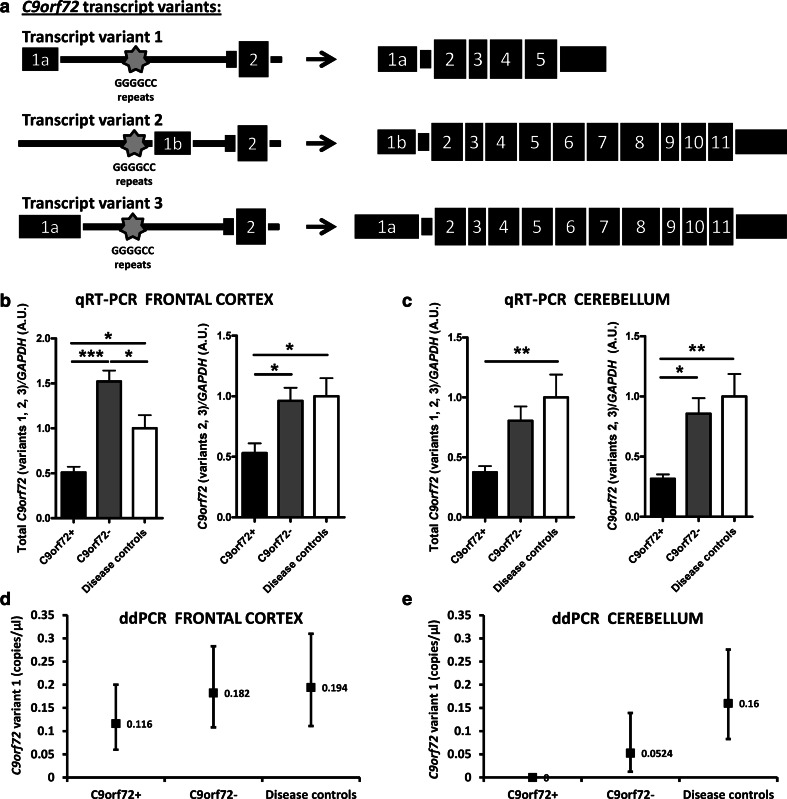
Frontal cortex and cerebellum tissue samples from the C9orf72+ group exhibit a significant decrease in *C9orf72* mRNA expression levels. **a** Schematic representation showing the three known transcript variants of *C9orf72*. The *star marks* the repeat location. **b**, **c** qRT-PCR for *C9orf72* transcript variants 1, 2, 3 and *C9orf72* transcript variants 2, 3 were performed in frontal cortex (**b**) and cerebellum (**c**). Data on graphs were normalized to disease control group (mean value set to 1). Statistical differences were calculated by one-way ANOVA with Tukey post hoc test. *p < 0.05, **p < 0.01, ***p < 0.005. **d**, **e** ddPCR was used to calculate absolute levels of the less abundant *C9orf72* transcript variant 1 in frontal cortex (**d**) and cerebellum (**e**). Transcript expression is expressed as number of copies per microliter. The mean expression and the range of expression across all samples tested are shown for each group. A clinical description of participants is available in Table 2 (online resource)

Given that brain tissue analysis of *C9orf72* mRNA expression levels has been limited to the frontal cortex [16, 22], we first sought to explore whether *C9orf72* mRNA expression is similarly decreased in the frontal cortex of our cohort of patients, and to evaluate whether a reduction is also observed in the cerebellum, another brain region affected in c9FTD/ALS [34]. We obtained brain tissue samples from ten ALS and FTD *C9orf72* repeat carriers (C9orf72+), nine ALS and FTD patients carrying a non-pathogenic *C9orf72* repeat (C9orf72−), and nine C9orf72− disease controls (clinical information in online resource, Table 2). We then isolated RNA from cerebella and frontal cortices and performed expression assays by quantitative real-time polymerase chain reaction (qRT-PCR) using Taqman probes targeting transcript variants 1, 2 and 3, as well as transcript variants 2 and 3 (Fig. 1a). As transcript variant 1 was difficult to detect through standard qRT-PCR, we assessed its expression using a highly precise and absolute nucleic acid quantification technique, termed droplet digital PCR (ddPCR) [25]. Moreover, we further compared expression levels obtained from standard qRT-PCR with ddPCR (online resource, Fig. 1, Table 1). We found that the C9orf72+ group exhibited decreased mRNA expression across all assays, as compared to normal repeat length carriers and disease control participants (Fig. 1b–e).

**Fig. 2:**
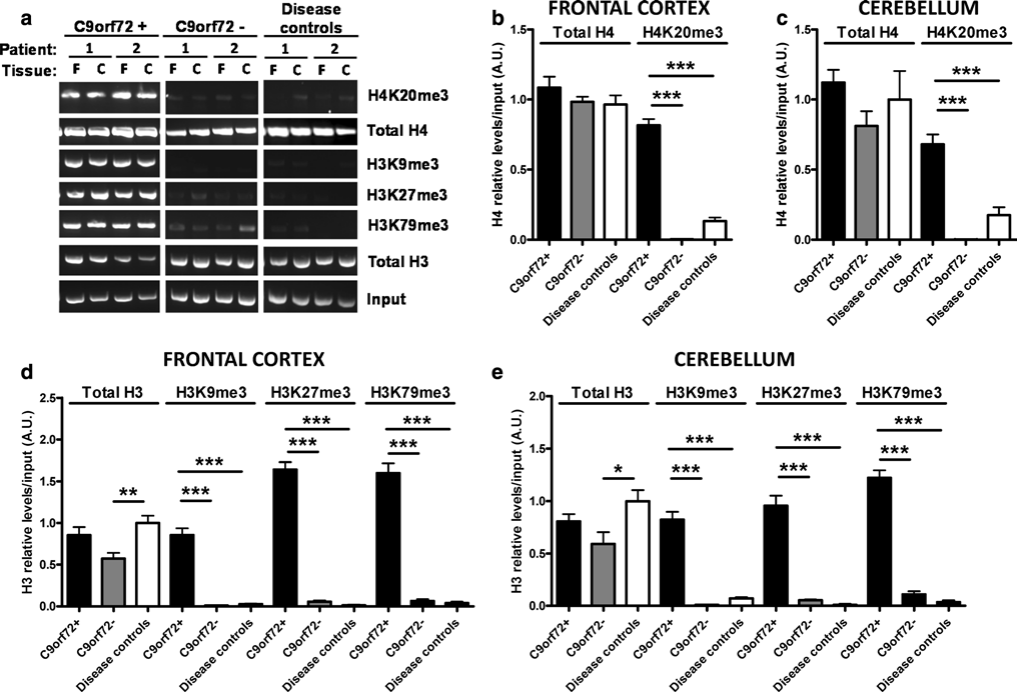
Reduced *C9orf72* mRNA expression levels in the C9orf72+ group result from aberrant binding to trimethylated histone residues. **a** Electrophoretic representation of chromatin immunoprecipitated DNA in a subgroup of participants tested. Chromatin immunoprecipitation (ChIP) was performed on two different human tissues, frontal cortex (F) and cerebellum (C), using antibodies specific for total histones H3 and H4 or trimethylated histones H3K9, H3K27, H3K79, and H4K20. Following pull-down, bound DNA was purified and used for PCR amplification of the *C9orf72* promoter region. This region was successfully amplified in the C9orf72+ group when ChIP was carried out with antibodies targeting trimethylated histone residues; under the same conditions, this region was not amplified in C9orf72− and disease controls, indicating an absence of binding. The complete figure is provided in the online resource. **b**, **c**, **d**, **e** Relative quantifications of all brain DNA were performed by measuring band intensity (complete gels in the online resource, Fig. 2) for each immunoprecipitated histone and presented as a ratio to the input. Each graph is normalized to total histone levels in the disease control group (mean value set to 1). Statistical differences were calculated by one-way ANOVA with Tukey post hoc test. *p < 0.05, **p < 0.01, *** p < 0.005. A clinical description of participants is available in Table 2 (online resource)

We next explored whether this decrease in *C9orf72* expression results from aberrant histone methylation, given that trimethylation of histone H3 at lysines 9, 27, 79, as well as of histone H4 at lysine 20, is linked to gene repression [3]. Using brain tissue from all 28 participants, we performed chromatin immunoprecipitation (ChIP) experiments using anti-H3K9me3, anti-H3K27me3, anti-H3K79me3, anti-(pan)H3 (total histone H3), as well as anti-H4K20me3 and anti-(pan)H4 (total histone H4). We successfully amplified the *C9orf72* promoter region in pathogenic repeat carriers, and found that the trimethylated residues only bind strongly to *C9orf72* repeat expansions (Fig. 2a–e). Of note, the *C9orf72* genomic region was amplified in all samples precipitated by both anti-(pan)H3 and anti-(pan)H4 (complete results in online resource, Fig. 2), confirming normal *C9orf72* binding to histones H3 and H4.

**Fig. 3:**
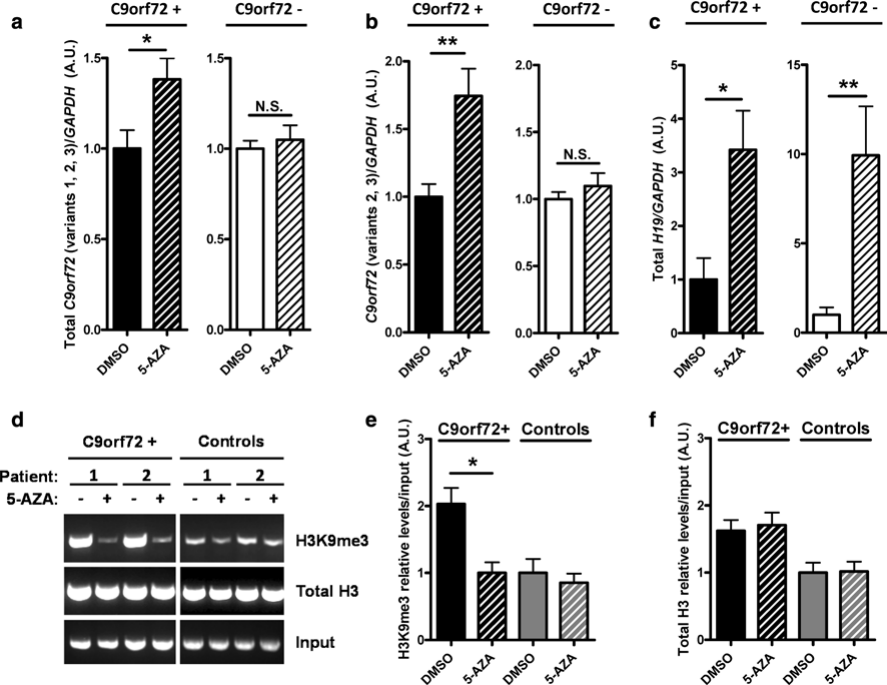
*C9orf72* mRNA expression is increased and H3K9me3 binding is decreased in C9orf72+ fibroblasts upon 5-AZA treatment. **a**, **b** qRT-PCR of RNA obtained from human fibroblasts grown in DMSO or 5-AZA demethylating agent. Both assays targeting transcript variants 1, 2, 3 (**a**) and 2, 3 (**b**) show a significant increase in expression after 5-AZA treatment only in *C9orf72* repeat expansion carriers. **c** qRT-PCR of *H19*, an imprinted gene, showing effectiveness of the 5-AZA treatment in C9orf72+ and C9orf72− fibroblasts. Statistical differences were assessed by unpaired Student *t* test. *p < 0.05, **p < 0.01. *NS* no significant difference. **d** Electrophoretic representation of chromatin immunoprecipitated DNA from a fibroblast subgroup using antibodies specific for total H3 or trimethylated histone H3K9. Fibroblasts were grown in DMSO or 5-AZA. Chromatin immunoprecipitation (ChIP) was performed in fibroblasts from C9orf72+ and C9orf72− participants. Following pull-down, bound DNA was purified and used for PCR amplification of the *C9orf72* promoter region. Upon treatment with vehicle (DMSO), the binding to trimethylated histone H3K9 in C9orf72+ cells, as assessed by the level of amplified *C9orf72* promoter region, was higher as compared to C9orf72−. Treatment with 5-AZA reduced this binding in C9orf72+ cases. The complete figure of all fibroblast lines is provided in the online resource. **e**, **f** Relative quantifications of all fibroblast lines were performed by measuring band intensity (complete gels in the online resource, Fig. 3) for each immunoprecipitated histone and presented as a ratio to the input. Graphs are normalized to total histone levels of disease control group (mean value set to 1). Statistical differences were calculated by one-way ANOVA with Tukey post hoc test. *p < 0.05. A clinical description of participants is available in Table 3 (online resource)

To confirm that reduced mRNA expression results from aberrant binding of expanded repeats to trimethylated lysine residues in c9FTD/ALS, we treated fibroblasts obtained from seven participants carrying normal alleles (C9orf72−) and seven patients carrying a (GGGGCC) repeat expansion (C9orf72+) (clinical information in online resource, Table 3) with 5-aza-2-deoxycytidine (5-AZA), a well-known DNA and histone demethylating agent [31, 33]. Following RNA isolation and reverse transcription, we conducted expression assays by qRT-PCR using probes targeting *C9orf72* transcript variants 1, 2 and 3, as well as transcript variants 2 and 3. Following 5-AZA treatment, *C9orf72* mRNA expression was significantly increased in fibroblasts from (GGGGCC) repeat expansion carriers, an effect not observed in fibroblasts from participants carrying normal alleles (Fig. 3a, b). However, mRNA expression of *H19*, an imprinted gene, was increased in both pathological and normal repeat carriers following 5-AZA treatment (Fig. 3c). Consistent with our brain ChIP experiments, we found that H3K9me3 strongly binds to *C9orf72* repeat expansions in DMSO-treated C9orf72+ fibroblasts but not in C9orf72− fibroblasts. Of importance, the 5-AZA-induced increase in *C9orf72* mRNA expression in C9orf72+ fibroblasts was accompanied by a decrease in binding of the *C9orf72* promoter to H3K9me3 (Fig. 3d, e). Similar to our studies using brain tissue, we found normal binding of *C9orf72* to histone H3 in all fibroblasts after precipitating samples with anti-(pan)H3 (Fig. 3f) (complete results in online resource, Fig. 3).

**Fig. 4:**
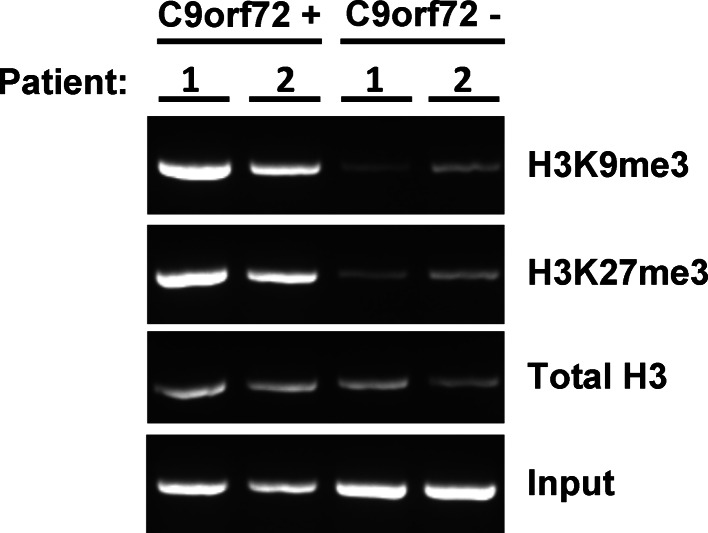
Strong binding of expanded *C9orf72* to H3K9me3 and H3K27me3 is detectable in the blood of c9FTD/ALS patients. Electrophoretic representation of chromatin immunoprecipitated blood DNA from two repeat expansion carriers (C9orf72+) and two participants carrying normal alleles (C9orf72−). Antibodies specific for total H3 or trimethylated histone H3K9 and H3K27 were used to pull-down DNA. Following pull-down, bound DNA was purified and used for PCR amplification of the *C9orf72* promoter region. The binding to trimethylated H3K9 and H3K27 in C9orf72+ blood cells, as assessed by the level of amplified *C9orf72* promoter region, was higher as compared to C9orf72−

Finally, to determine whether these biological changes are detectable in peripheral blood, we isolated mononuclear cells from blood collected from two repeat expansion carriers (C9orf72+) and two participants carrying normal alleles (C9orf72−), and conducted ChIP experiments using anti-H3K9me3, anti-H3K27me3, and anti-(pan)H3. As in the brain, we confirmed that only mutant *C9orf72* strongly binds H3K9me3 and H3K27me3, despite normal binding of the *C9orf72* promoter region to histone H3 in all four samples (Fig. 4).

Our experiments provide compelling evidence that repression of the *C9orf72* gene in pathogenic (GGGGCC) repeat carriers is caused by the binding of mutant *C9orf72* to trimethylated lysine residues within histones H3 and H4.

## Discussion

In this study, we show that the decrease in *C9orf72* expression levels in brain tissue and fibroblasts derived from pathogenic *C9orf72* expansion carriers is associated with enhanced binding of mutant *C9orf72* to trimethylated lysine residues within histones H3 and H4. We also demonstrate that these epigenetic changes are detectable in peripheral blood, a finding that, when confirmed in a larger cohort of patients, may represent a disease-specific biomarker of c9FTD/ALS.

**Fig. 5:**
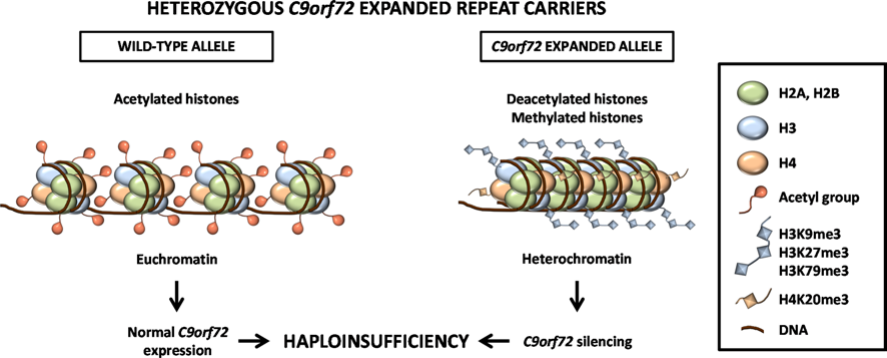
Schematic representation of the proposed haploinsufficiency mechanism resulting from epigenetic changes in c9FTD/ALS

Our data suggest that the decrease in *C9orf72* mRNA expression results from heterochromatin formation following aberrant histone methylation. As *C9orf72* expansions are mostly inherited in an autosomal dominant fashion, heterozygous *C9orf72* expanded repeat carriers carry both a wild-type transcriptionally active allele (euchromatin), as well as a mutated transcriptionally silent allele (heterochromatin). The wild-type allele is most likely unable to produce enough C9orf72 protein to compensate for the loss of protein from the mutated allele, thereby leading to haploinsufficiency (Fig. 5). As histone methylation is commonly perceived as inducing flexible short-term gene silencing through regulation of numerous histone-modifying complexes, regulation of the chromatin structure varies depending on the availability of chromatin remodeling enzymes, including histone acetyltransferases, histone deacetylases, histone methyltransferases and histone demethylases. This dynamic chromatin structure likely influences transcription of expanded *C9orf72*, which consequently might either accumulate as RNA foci or translate into aggregation-prone c9RAN proteins.

While our studies were in progress, the Rogaeva’s group reported DNA hypermethylation of the upstream CpG island in about 40 % of all ALS cases that were tested [43]. Although they found no hypermethylation in about 60 % of the ALS cases studied, it remains possible that another CpG island located farther away from the repeat might be methylated in these patients. Several CpG sites within the repeat itself may also be methylated, as suggested by the methyl-sensitive restriction enzyme digest and sequencing (MRE-seq) CpG score tool (http://genome.ucsc.edu). Of note, the number of CpG sites in the repeat region is increased in expansion carriers, creating a new CpG island that can be a target for methylation. Future studies will need to determine whether the expanded repeat as well as nearby CpG islands are methylated, and estimate the frequency of aberrant DNA methylation in both ALS and FTD cases. While DNA methylation is commonly believed to trigger histone methylation, we now know that methylation of either DNA or histone residues can appear first, leading to aberrant methylation of the other structure [10]. If DNA methylation appears first, it must happen well before the onset of symptoms; increased binding of mutant *C9orf72* to trimethylated histone H3K9 was similarly present in two asymptomatic repeat carriers (28 and 30 years of age) and in patients already diagnosed with ALS or FTD (clinical information in online resource, Table 3). The expanded repeat may also lead to an unusual DNA structure, increasing DNMTs’ capacity to catalyze methyl groups to cytosine residues within the repeat itself. This aberrant DNA methylation in turn may trigger methylation of histones H3 and H4. Regardless of which structure becomes methylated first, our findings support the notion that it is the aberrant methylation of histones H3 and H4 residues in all c9FTD/ALS patient samples evaluated that caused the decreased expression of *C9orf72* mRNA.

The expanded non-coding (GGGGCC) repeat, which is located between two alternatively spliced non-coding 5′ exons of *C9orf72*, produces three distinct transcript variants (Fig. 1a). Transcript variants 1 (NM_145005.5) and 3 (NM_001256054.1) have the repeat located in the intronic region and have been suggested to lead to RNA foci formation and RNA-binding protein sequestration, while the expression of variant 2 (NM_018325.3), with the repeat located in the promoter region, has been shown to be silenced in pathogenic repeat carriers [16, 29]. Others reported reduced expression of the three known transcript variants [22]. Given this difference in repeat location and considering the involvement of epigenetic changes, the transcription level of each variant may not be uniformly altered by methylation, thereby resulting in different disease courses and/or biological outcomes. Such methylation variability has previously been shown to strongly influence phenotypes in Fragile X and Rett syndromes [8, 40]. Future studies will need to evaluate whether methylation variability similarly influence ALS and FTD phenotypes.

Our results rise important questions regarding the mid-adulthood onset of disease, as the methylation pattern of histones H3 and H4 varies with age, increasing or decreasing depending on histone residues [11]. Of interest, the trimethylated histone residues aberrantly bound by mutant *C9orf72* are also known to be methylated in an age-dependent manner. Specifically, methylation at residue H3K9 decreases with age, and methylation at residues H3K27, H3K79 and H4K20 increases [17, 38, 42]. Moreover, it is well-known that accumulation of reactive oxygen species over time leads to an exponential increase in molecular oxidative stress, an event extensively associated with ALS [12], which has been suggested to cause a gradual decline in homeostatic mechanisms associated with aging [5, 39]. It has also been suggested that epigenetic mechanisms resulting from aging possibly play an important physiopathological role in the presence of oxidative stress [11]. Oxidative stress is known to alter the balance between acetylation/deacetylation and methylation/demethylation processes in histone residues, deregulating the expression of pro-inflammatory genes. As such, aberrant histone methylation as a result of *C9orf72* repeat expansions, oxidative stress accumulation leading to epigenetic deregulation, and normal age-related epigenetic mechanism variations could all contribute to the adult onset of the disease as well as the neuroinflammation found in ALS patients [44]. Since c9FTD/ALS histone-associated methylation can be easily detected using patient blood, it will be of interest to evaluate whether aberrant histone methylation changes over the course of the disease.

Our results suggest that c9FTD/ALS can now be considered among the class of repeat disorders marked by chromatin modifications, such as myotonic dystrophy, SCA types 8 and 31, Friedreich ataxia, Fragile X and Fragile X-associated tremor/ataxia syndromes [6]. Appropriate chromatin conformation in neurons is essential to prevent DNA damage, to assure proper DNA packaging and to maintain normal gene expression. Similar to myotonic dystrophy and SCA8, three distinct pathogenic processes appear to be associated with c9FTD/ALS pathogenesis. First, *C9orf72* repeat expansions lead to the formation of RNA foci, which have the potential to sequester RNA-binding proteins and impair their function [16, 29]. Second, we and others have recently reported that *C9orf72* repeat expansions lead to RAN translation resulting in the production of poly-glycine–alanine, poly-glycine–arginine and poly-glycine–proline peptides that accumulate as insoluble aggregates in the cytoplasm of neurons [2, 30], further supporting an RNA-mediated mechanism of toxicity in c9FTD/ALS. Third, our current study suggests that binding of mutant *C9orf72* to trimethylated lysine residues within histones H3 and H4 causes repression of the *C9orf72* gene, an event that may play an important role in the development of c9FTD/ALS. Taken together, early studies suggest a C9orf72 loss of function, both through neurotoxic processes and epigenetic changes, as a disease mechanism, and demonstrate that a better understanding of the C9orf72 protein function is crucial in order to identify therapeutic targets and to develop effective treatment strategies [21]. Additional research to examine DNA and histone methylation as a part of c9FTD/ALS pathogenesis may also lead to the identification of novel therapeutic targets for ALS and FTD. The need for such work is urgent: despite an enormous effort to elucidate the molecular mechanisms involved in FTD and ALS, the prognosis for patients has not improved, and no effective treatment has been developed. The discovery that *C9orf72* haploinsufficiency contributes to disease pathogenesis and that epigenetic processes mediate this effect offers a unique opportunity for therapeutic development, providing c9FTD/ALS patients with new hope.

## Electronic supplementary material

Below is the link to the electronic supplementary material.

Supplementary material 1 (DOCX 10151 kb)
