# Supplementary material for: Reduced C9orf72 gene expression in c9FTD/ALS is caused by histone trimethylation, an epigenetic event detectable in blood
Source: Acta Neuropathol. 2013 Oct 29;126(6):895–905. doi: 10.1007/s00401-013-1199-1 (PMC3830740; doi:10.1007/s00401-013-1199-1)
Supplement: Supplementary file 1 — Supplementary material 1 (DOCX 10151 kb) [file 401_2013_1199_MOESM1_ESM.docx]

**Online resource**

**Title:** Reduced *C9orf72* gene expression in c9FTD/ALS is caused by histone trimethylation, an epigenetic event detectable in blood.

**Journal name:** Acta Neuropathologica

**Authors:** Veronique V. Belzil, Ph.D., Peter O. Bauer, M.D., Ph.D., Mercedes Prudencio, Ph.D. , Tania F. Gendron, Ph.D. , Caroline T. Stetler, M.A., Irene K. Yan, Luc Pregent, Lillian Daughrity, Matthew C. Baker, Rosa Rademakers, Ph.D., Kevin Boylan, M.D., Dennis W. Dickson, M.D., Tushar C. Patel, M.D. , and Leonard Petrucelli, Ph.D.

**Corresponding author:**

Leonard Petrucelli, Ph.D.

Department of Research, Neuroscience

Mayo Clinic College of Medicine

4500 San Pablo Road

Jacksonville, FL 32224

Office: 1-904-953-2855

Fax: 1-904-953-6276

E-mail: [petrucelli.leonard@mayo.edu](mailto:petrucelli.leonard@mayo.edu)

**Figure 1**

**Fig. 1**

*C9orf72* mRNA expression levels measured by ddPCR confirms qRT-PCR results. RNA isolated from frontal cortex and cerebellum of C9orf72+, C9orf72-, and disease control groups (see Table 2 for clinical information on participants) was analyzed for RNA integrity. ddPCR was used to calculate absolute levels of *C9orf72* transcript variants 1, 2, 3 (**a, b**) and transcript variants 2, 3 (**c, d**) in frontal cortex (**a**, **c**) and cerebellum (**b**, **d**). mRNA expression is expressed as the number of copies per microliter. The mean expression, and the range of expression across all samples tested are shown for each group. Both frontal cortex (**a, c**) and cerebellum results (**b, d**) show decreased *C9orf72* expression levels in C9orf72+ cases, consistent with qRT-PCR results (see Fig. 1b-e).

**Table 1**

Number of *C9orf72* copies detected by ddPCR.

|  | **FRONTAL CORTEX** | | | **CEREBELLUM** | | |
| --- | --- | --- | --- | --- | --- | --- |
|  | **C9orf72** | **SE max** | **SE min** | **C9orf72** | **SE max** | **SE min** |
|  | **Transcript variant 1 (Fig.1 d,c)** | | | | | |
| **C9orf72+** | 0.116 | 0.084 | 0.056 | 0 | 0 | 0 |
| **C9orf72-** | 0.182 | 0.101 | 0.074 | 0.0524 | 0.0866 | 0.04 |
| **Disease controls** | 0.194 | 0.116 | 0.083 | 0.16 | 0.116 | 0.0772 |
|  | **Transcript variants 1,2,3 (online resource Fig.1 a,b)** | | | | | |
| **C9orf72+** | 31.4 | 10.1 | 9.2 | 28.7 | 8.9 | 10.7 |
| **C9orf72-** | 41 | 18.3 | 18.4 | 35.2 | 1.1 | 1.2 |
| **Disease controls** | 34.3 | 1.3 | 1.3 | 32.4 | 1.1 | 1.1 |
|  | **Transcript variant 1,2 (online resource Fig. 1 c,d)** | | | | | |
| **C9orf72+** | 18.2 | 0.9 | 0.8 | 28.7 | 8.9 | 10.7 |
| **C9orf72-** | 33.4 | 1.3 | 1.3 | 35.2 | 1.1 | 1.2 |
| **Disease controls** | 21.8 | 1.1 | 1 | 32.4 | 1.1 | 1.1 |

**Figure 2**

ChIP experiment for all brain tissue samples. ChIP analyses were performed on two different human tissues: frontal cortex (F) and cerebellum (C). Vertical white lines denote limits of electrophoretic gels. A subgroup of this experiment is included in Fig. 2a using cases 5 and 6 for C9orf72+ group, and cases 3 and 4 for both C9orf72- and disease control groups.

**Figure 3**

ChIP experiment for all participants-derived fibroblasts. ChIP analyses were performed using human fibroblasts derived from either *C9orf72* expanded repeat carriers or participants carrying normal alleles, and treated with DMSO or 5-AZA. Vertical white lines denote limits of electrophoretic gels. A subgroup of this experiment is included in Fig. 3d using cases 6 and 7.

**Table 2**

Genetic and clinical information of brain study participants. *C9orf72*+, *C9orf72*- and control cases were selected based on sex, age, and tissue availability from The Mayo Clinic Jacksonville Brain Bank

| Group | Sample # | Patient ID # | Genotype | Pathological  diagnosis | Sex | Age of onset (years) | Duration (months) |
| --- | --- | --- | --- | --- | --- | --- | --- |
| c9FTD/ALS  *C9orf72+* | 1 | 00-124 | *C9orf72* expansion | ALS/PA | F | 61 | 33 |
|  | 2 | 03-223 | *C9orf72* expansion | ALS | M | 56 | 26 |
|  | 3 | 99-211 | *C9orf72* expansion | FTLD-U/HpScl/PA | M | 62 | 131 |
|  | 4 | 10-115 | *C9orf72* expansion | FTLD/MND/HpScl | M | <60 | 60 |
|  | 5 | 11-306 | *C9orf72* expansion | FTLD-U/HpScl | M | 68 | 71 |
|  | 6 | 10-031 | *C9orf72* expansion | ALS | M | 52 | 76 |
|  | 7 | 13-018 | *C9orf72* expansion | ALS | M | 49 | 49 |
|  | 8 | 07-244 | *C9orf72* expansion | FTLD-U/HpScl | M | 67 | 98 |
|  | 9 | 08-362 | *C9orf72* expansion | FTLD-U | M | 70 | 28 |
|  | 10 | 09-306 | *C9orf72* expansion | FTLD-U | M | 74 | 93 |
| FTD/ALS *C9orf72*- | 1 | 09-403 | Normal *C9orf72* repeat size | ALS/PA | F | 60 | 15 |
|  | 2 | 10-136 | Normal *C9orf72* repeat size | ALS/heterotopia | M | 45 | 58 |
|  | 3 | 11-067 | Normal *C9orf72* repeat size | ALS | M | 50 | 47 |
|  | 4 | 12-355 | Normal *C9orf72* repeat size | ALS/SC | M | 53 | 65 |
|  | 5 | 00-138 | Normal *C9orf72* repeat size | FTLD-U/HpScl/AGD | M | 63 | 51 |
|  | 6 | 02-144 | Normal *C9orf72* repeat size | FTLD-U/HpScl/AGD/VaD | M | 73 | 105 |
|  | 7 | 06-149 | Normal *C9orf72* repeat size | FTLD-U/HpScl/PA/BLBD | M | 74 | 45 |
|  | 8 | 06-301 | Normal *C9orf72* repeat size | FTLD-U/VaD/AGD | M | 65 | 18 |
|  | 9 | 07-269 | Normal *C9orf72* repeat size | FTLD-U/Limbic gliosis | M | 66 | 122 |
| Disease controls | 1 | 01-183 | Normal *C9orf72* repeat size | PA/CVA | M | 72 | 112 |
|  | 2 | 03-259 | Normal *C9orf72* repeat size | PA/VaD | F | n/a | n/a |
|  | 3 | 06-005 | Normal *C9orf72* repeat size | AGD/PA | F | 82 | 22 |
|  | 4 | 06-183 | Normal *C9orf72* repeat size | AGD/Fahr | M | 72 | 50 |
|  | 5 | 07-279 | Normal *C9orf72* repeat size | Normal | F | 64 | 9 |
|  | 6 | 09-001 | Normal *C9orf72* repeat size | VaD | M | 63 | 79 |
|  | 7 | 09-107 | Normal *C9orf72* repeat size | Globus pallidus ischemia | F | 63 | 177 |
|  | 8 | 10-041 | Normal *C9orf72* repeat size | VaD/HpScl | M | 63 | 100 |
|  | 9 | 10-253 | Normal *C9orf72* repeat size | VaD/SC | M | unavailable | unavailable |

AGD: Argyrophilic Grain Disease, ALS: Amyotrophic Lateral Sclerosis, BLBD: Brainstem Lewy Body Disease, CVA: Cerebral Vascular Accident, Fahr: Fahr’s Syndrome, FTLD-U: Frontotemporal Lobar Degeneration with Ubiquitin inclusions, HpScl: Hippocampal Sclerosis, ILBD: Incidental Lewy Body Disease, MND: Motor Neuron Disease, PA: Pathological Aging, SC: Sydenham’s chorea, VaD: Vascular Dementia. M: male, F: female.

**Table 3**

Genetic and clinical information of fibroblast study participants.

| Sample ID # | Genotype | Phenotype | Sex | Age of onset (years) | Duration |
| --- | --- | --- | --- | --- | --- |
| 1058 | *C9orf72* expansion | ALS | F | 43 | Still alive after 1 year |
| 001-01 | *C9orf72* expansion | ALS | M | 46 | Still alive after 3 years |
| 002-14 | *C9orf72* expansion | ALS/FTD | F | 63 | Still alive after 2 years |
| 002-19 | *C9orf72* expansion | Healthy at 30 years old | F | n/a | n/a |
| 002-20 | *C9orf72* expansion | Healthy at 28 years old | F | n/a | n/a |
| 003-01 | *C9orf72* expansion | ALS | F | 49 | Still alive after 1 year |
| 005-01 | *C9orf72* expansion | ALS/FTD | M | 41 | Still alive after 2 years |
| 002-02 | Normal *C9orf72* repeat size | sixth nerve palsy | F | n/a | n/a |
| 003-03 | Normal *C9orf72* repeat size | Family control, healthy | F | n/a | n/a |
| 004-01 | Normal *C9orf72* repeat size  *TARDBP* p.A315T mutation | ALS | M | 51 | Still alive after 3 years |
| 006-01 | Normal *C9orf72* repeat size  Negative for *SOD1*, *TARDBP*, *FUS* mutation | ALS/FTD | M | 55 | Still alive after 8 years |
| 007-01 | Normal *C9orf72* repeat size | Progressive muscular atrophy | M | 70 | Still alive after 3 years |
| 008-01 | Normal *C9orf72* repeat size  Negative for *SOD1*, *TARDBP*, *FUS* mutation | ALS | F | 62 | Still alive after 2 years |
| iHCA | Normal *C9orf72* repeat size | Healthy control | F | n/a | n/a |

ALS: Amyotrophic Lateral Sclerosis, FTD: Frontotemporal Dementia. M: male, F: female. Note: cell shading indicates members of the same family.

**Table 4**

Genetic and clinical information of blood study participants.

| Sample ID # | Genotype | Phenotype | Sex | Age of onset (years) | Duration |
| --- | --- | --- | --- | --- | --- |
| 015-01 | *C9orf72* expansion | ALS | M | 49 | Recently diagnosed |
| 016-01 | *C9orf72* expansion | ALS | F | 62 | Still alive after 1 year |
| 014-01 | Normal *C9orf72* repeat size  Negative for *SOD1*, *TARDBP*, *FUS* mutation | ALS | F | 69 | Still alive after 4 years |
| 500-01 | Normal *C9orf72* repeat size | ALS/PD | M | 50 | Still alive after 4 years |

ALS: Amyotrophic Lateral Sclerosis, PD: Parkinson’s disease. M: male, F: female.
